# Replication mechanisms of circular ssDNA plant viruses and their potential implication in viral gene expression regulation

**DOI:** 10.1128/mbio.01692-23

**Published:** 2023-09-11

**Authors:** Mélia Bonnamy, Stéphane Blanc, Yannis Michalakis

**Affiliations:** 1 PHIM, Univ Montpellier, IRD, CIRAD, INRAE, Institut Agro, Montpellier, France; 2 MIVEGEC, CNRS, IRD, Univ Montpellier, Montpellier, France; Albert Einstein College of Medicine, Bronx, New York, USA

**Keywords:** viruses, single-stranded DNA viruses, rolling-circle replication, recombination-dependent replication, replication, copy number variation, gene copy number, *Geminiviridae*, *Nanoviridae*, genome formula

## Abstract

The replication of members of the two circular single-stranded DNA (ssDNA) virus families *Geminiviridae* and *Nanoviridae*, the only ssDNA viruses infecting plants, is believed to be processed by rolling-circle replication (RCR) and recombination-dependent replication (RDR) mechanisms. RCR is a ubiquitous replication mode for circular ssDNA viruses and involves a virus-encoded Replication-associated protein (Rep) which fulfills multiple functions in the replication mechanism. Two key genomic elements have been identified for RCR in *Geminiviridae* and *Nanoviridae*: (i) short iterative sequences called iterons which determine the specific recognition of the viral DNA by the Rep and (ii) a sequence enabling the formation of a stem-loop structure which contains a conserved motif and constitutes the origin of replication. In addition, studies in *Geminiviridae* provided evidence for a second replication mode, RDR, which has also been documented in some double-stranded DNA viruses. Here, we provide a synthesis of the current understanding of the two presumed replication modes of *Geminiviridae* and *Nanoviridae*, and we identify knowledge gaps and discuss the possibility that these replication mechanisms could regulate viral gene expression through modulation of gene copy number.

## INTRODUCTION

Replication is a critical step in the life cycle of viruses as it primarily permits to generate progeny that will contribute to virus spread. One important category of viral replication is that of the single-stranded DNA (ssDNA) viruses, which do not involve virus-encoded polymerases but viral replication-associated proteins referred as Rep (1). DNA replication controlled by Rep proteins has been reported for bacterial plasmids, and an immense paraphyletic group of small ssDNA viruses infecting procaryotes and all sorts of eucaryotes, including invertebrates, vertebrates, and plants (2). Whether and how the replication process can fulfill additional functions, other than progeny production, is an issue that we illustrate through some speculations resulting from the survey of the replication mechanisms in the two families *Geminiviridae* and *Nanoviridae* that encompass all currently known ssDNA viruses infecting plants.

Members of the family *Geminiviridae* (geminiviruses) have a highly diversified host range, infecting both monocotyledons and dicotyledons. They are responsible for considerable losses on crops of major socio-economic interest to many countries, particularly in tropical and sub-tropical zones, such as maize, wheat, sugar cane, cassava, cucurbits, sweet potatoes, pepper, tomato, and cotton. Their worldwide dispersal has been facilitated by human activities and mobility, agricultural intensification, and global warming expanding the geographical range of their vectors (3). Epidemics of new emerging geminiviruses are frequent (4). Recently, the emergence of new species, infecting perennial crops of major socio-economic interest also in temperate regions, including grapes, has drawn particular attention (5, 6). The appearance of new species is due, in part, to their high rate of mutation, recombination, and reassortment, making this virus family one of the most diverse (3). It is, therefore, of paramount interest to understand the replication processes generating such a genetic diversity.

**Fig 1:**
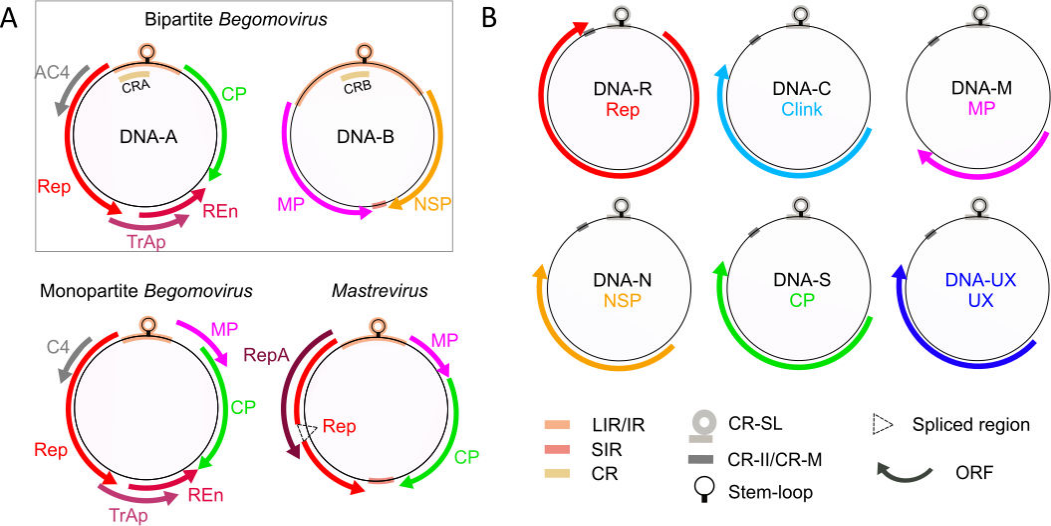
Genome organization of the families *Geminiviridae* and *Nanoviridae*. (**A**) Genome organization in the genera *Begomovirus* and *Mastrevirus* belonging to the family *Geminiviridae. Geminiviridae* are classified into 14 genera, which can be found in reference 7. All *Geminiviridae* genera have a genome constituted by a single DNA molecule (black circles) except for the genus *Begomovirus* which contains species that can be either monopartite or bipartite with complementary DNA-A and DNA-B. Solely, the genomes of begomoviruses and mastreviruses are shown here because most of the experimental work we review has been obtained with these virus models. (**B**) Genome organization in the family *Nanoviridae*. The name of each component is indicated within the corresponding circle. The five genomic components DNA-R, C, M, N, and S are identified for species of both genera *Nanovirus* and *Babuvirus*. DNA-UX represents components for which no function has so far been determined. The genus *Nanovirus* possesses three components of unknown function named DNA-U1, DNA-U2, and DNA-U4. The genus *Babuvirus* possesses only one component of unknown function named DNA-U3 (12). The colored arrows around the circles represent the ORF. For *Geminiviridae* genomes, arrow orientation reflects the position on the viral or complementary strand, and alternatively spliced regions are indicated by dotted triangles. Intergenic and conserved non-coding regions are represented by rectangles inside or overlapping the black circles, and their identity is indicated below the panel. At the top of each DNA circle, a highly conserved stem-loop structure is represented. Rep: replication-associated protein; repA and REn: replication enhancer proteins; TrAp: transcriptional activator protein; CP: capsid protein; MP: movement protein; NSP: nuclear shuttle protein; AC4/C4: protein with multiple functions; U1, U2, U3, and U4 (UXs): proteins with unknown functions; LIR: long intergenic region; SIR: small intergenic region; CRA and CRB: common regions of DNA-A and DNA-B corresponding to the highly conserved 200-nucleotide stretch within the large intergenic region of bipartite begomoviruses ; CR-SL: common region-stem loop; CR-II: second common region (*Nanovirus*); CR-M: major common region (*Babuvirus*) (7, 12).

The family *Geminiviridae* is composed of 14 genera (Fig. 1A) (7). Most geminiviruses have their genetic information distributed over one single-stranded circular DNA molecule. However, members of the genus *Begomovirus* can be mono- or bi-partite with their genome constituted either by one single DNA circle or by two complementary circles named DNA-A and DNA-B. For both mono- and bipartite species, the virus particle is a geminate structure resulting from the fusion of two icosahedral “subunits,” and each geminate particle contains a single DNA circle of comparable size ranging from 2.5 to 3.2 kb (8, 9). DNA-A and DNA-B of bipartite begomoviruses are, therefore, encapsidated in two distinct geminate virus particles. Each circular ssDNA molecule is composed of coding and non-coding regions (Fig. 1A). Coding regions are transcribed bidirectionally, and the number of open reading frames (ORF) differs according to the geminivirus genus (7, 10). The number and the name of the intergenic non-coding regions also vary depending on the genus (Fig. 1A) (7). Specifically, for the bipartite begomoviruses, the non-coding intergenic region (IR) contains a sequence of about 200 nucleotides defined as the conserved region (CR), that is, identical between DNA-A and DNA-B (11). In all genera, the non-coding regions contain regulatory sequences for replication and expression.

The family *Nanoviridae* (nanovirids), with their small number of identified species and restricted host range, have long been considered a minor threat to agriculture (13). As a result, they have received little attention from the scientific community, whose research has tended to focus on geminiviruses. The *Banana bunchy top virus* (BBTV) is an exception, however, as it is responsible for banana bunchy top disease, one of the oldest known viral diseases of plants and considered to be the most destructive viral disease for the world agriculture (14). In recent years, epidemics due to the emergence of new nanoviruses have broadened the host spectrum, and thus the threat, to crops of prime importance for many countries, and above all numerous legume crops (13).

The family is thus far composed of solely two genera *Nanovirus* and *Babuvirus* (Fig. 1B). It is characterized by a multipartite genome architecture, where the genetic information is borne by eight (*Nanovirus*) or six (*Babuvirus*) ssDNA circular molecules called genome segments or components. Each component is individually encapsidated, all are about 1 kb long, and all encode a single protein and comprise a non-coding regulatory region. In all cases, the ORFs are unique and transcribed unidirectionally in the virion-sense (15 – 18). The only known exception to this rule is for the BBTV DNA-R, where a second ORF coding for a small protein with unknown function has been identified and nested within the Rep ORF (19). Non-coding regions contain two conserved sequences, respectively, called common region stem-loop (CR-SL) and second common region (CR-M for *Babuvirus* or CR-II for *Nanovirus*) (Fig. 1B) (12). As for geminiviruses, the non-coding regions contain regulatory sequences for replication and expression.

While the replication of geminiviruses has been extensively studied, the replication of nanovirids has been little explored. Because these two families share some genomic similarities, most of the available information concerning nanovirids replication is either assumed by analogy or has been inferred by homology with geminiviruses. It is established that both geminiviruses and nanovirids genomes are replicated according to the rolling-circle replication (RCR) (20, 21), a mechanism that has also been described for the replication of all the other eukaryotic circular ssDNA viruses (22), some bacterial plasmids (23), and some single- and double-stranded DNA (dsDNA) phages (24). A series of studies conducted on geminiviruses suggested the existence of another mode of replication, also reported in some phages, the recombination-dependent replication (RDR). Here, we survey the current knowledge on the replication mechanisms of geminiviruses and nanovirids. We propose schematic representations for the different replication modes they may use. Importantly, we also highlight the fragmentary nature of our knowledge on this issue: (i) there is no single species where all steps of the replication cycle have been studied and the complete picture stems from inferences across species (Table 1), (ii) the quantitative importance of RCR versus RDR is unknown and the fate of replicative-intermediates is unclear, and (iii) the differential accumulation of genomic segments in multipartite species is not fully explained by the currently known mechanisms.

**TABLE 1:** Survey of RCR studies in *Geminiviridae* and *Nanoviridae*

Step	Special feature	Geminiviridae			Nanoviridae		
		Species	Genus	Reference	Species	Genus	Reference
Complementary-strand synthesis	DNA primers	MSV	*Mastrevirus*	(25, 26)	BBTV	*Babuvirus*	(27)
		DSV		(28)			
		CSMV		(29)			
		WDV		(30)			
		TobYDV		(31)			
	RNA primers	ACMV	*Begomovirus* (bipartite)	(32)	N/I^*a*^	N/I	N/I
	Involvement of a host primase	TYLCV	*Begomovirus* (monopartite)	(33)	N/I	N/I	N/I
		ToLCNDV	*Begomovirus* (bipartite)	(34)			
	Complementary-strand synthesis involves host polymerase α	TYLCV	*Begomovirus* (monopartite)	(35)	N/I	N/I	N/I
Viral DNA recognition	Identification of iteron sequences on the right side of the stem-loop sequence only	Several species	Several genera	(36)	N/I	N/I	N/I
		TGMV	*Begomovirus* (bipartite)	(37 – 39)			
		BGMV	*Begomovirus* (bipartite)	(39)			
		SqLCV	*Begomovirus* (bipartite)	(38)			
		BCTV	*Curtovirus*	(40, 41)			
		TYLCV	*Begomovirus* (monopartite)	(42, 43)			
		ToLCV-Nde	*Begomovirus* (bipartite)	(44)			
		ToMoTV	*Begomovirus* (bipartite)	(45)			
	Identification of iteron sequences on both sides of the stem-loop sequence	WDV	*Mastrevirus*	(46, 47)	FBNYV	*Nanovirus*	(48, 49)
					MDV	*Nanovirus*	(49)
		MSV	*Mastrevirus*	(50)	SCSV	*Nanovirus*	(49)
					BBTV	*Babuvirus*	(51, 52)
		MYMIV	*Begomovirus* (bipartite)	(53)	ABTV	*Babuvirus*	(52)
					CBDV	*Babuvirus*	(52)
	Rep recognizing the common region/iterons of the viral genome in a sequence-specific manner	TGMV	*Begomovirus* (bipartite)	(37 – 39, 54 – 58)	FBNYV	*Nanovirus*	(49)
		BGMV	*Begomovirus* (bipartite)	(39, 58)	MDV	*Nanovirus*	(49)
		SqLCV	*Begomovirus* (bipartite)	(38)	SCSV	*Nanovirus*	(49)
		BCTV	*Curtovirus*	(40, 41)	BBTV	*Babuvirus*	(51)
		TYLCV	*Begomovirus* (monopartite)	(42, 43)			
		WDV	*Mastrevirus*	(46, 47, 59)			
		ToLCV-Nde	*Begomovirus* (bipartite)	(44, 60)			
		MSV	*Mastrevirus*	(50)			
		MYMIV	*Begomovirus* (bipartite)	(53)			
	Rep oligomerization	TGMV	*Begomovirus* (bipartite)	(55, 61)	N/I	N/I	N/I
	The stem-loop sequence is not involved in recognition specificity of the viral DNA by the corresponding Rep protein	TGMV	*Begomovirus* (bipartite)	(39)	N/I	N/I	N/I
		BGMV	*Begomovirus* (bipartite)	(39)			
Replication initiation	Conserved stem-loop sequence is required for replication	TGMV	*Begomovirus* (bipartite)	(39, 62, 63)	BBTV	*Babuvirus*	(64)
		SqLCV	*Begomovirus* (bipartite)	(38)			
		MSV	*Mastrevirus*	(65)			
		MDV	*Mastrevirus*	(66)			
		BGMV	*Begomovirus* (bipartite)	(39)			
		TYLCV	*Begomovirus* (monopartite)	(67)			
		WDV	*Mastrevirus*	(59)			
	Rep binding leads to a distortion of the DNA structure at the origin of replication	MYMIV	*Begomovirus* (bipartite)	(53)	N/I	N/I	N/I
	Rep cleavage in the nonanucleotide sequence	ACMV	*Begomovirus* (bipartite)	(68)	BBTV	*Babuvirus*	(64)
		TYLCV	*Begomovirus* (monopartite)	(67, 69)	FBNYV	*Nanovirus*	(48)
		WDV	*Mastrevirus*	(69, 70)			
New strand elongation	Rep protein remains covalently linked to the 5′-end of the cleaved positive single strand	TYLCV	*Begomovirus* (monopartite)	(67)	BBTV	*Babuvirus*	(64)
	Rep helicase activity	TYLCSV	*Begomovirus* (monopartite)	(71)	N/I	N/I	N/I
		MYMV	*Begomovirus* (bipartite)	(72)			
		MYMIV	*Begomovirus* (bipartite)	(72)			
		ICMV	*Begomovirus* (bipartite)	(72)			
	New strand synthesis is performed by host polymerase δ or ε	TYLCV	*Begomovirus* (monopartite)	(35)	N/I	N/I	N/I
Joining of the plus-strand extremities	Rep cleavage and joining activity	TYLCV	*Begomovirus* (monopartite)	(67, 69)	BBTV	*Babuvirus*	(64)
		WDV	*Mastrevirus*	(69)			

^*a*^ N/I: not investigated.

## ROLLING CIRCLE REPLICATION

### Geminiviruses

Geminiviruses replication takes place in the nucleus of host cells (73), where they boost and divert the host DNA synthesis machineries. Some geminiviruses are restricted to phloem vascular tissues, while others are also present in mesophyll cells (74 – 76). All these cell types are fully differentiated and have therefore left the replicative stage of the cell cycle, implying that some factors required for viral DNA synthesis/replication are no longer produced. Geminiviruses can at least partially reprogram the cell cycle of their host in order to re-induce the replicative phase of the cell cycle and benefit from cellular actors necessary for DNA replication (73, 77, 78). This partial reprogramming is mediated by the interaction of geminiviral proteins with components of the cell cycle regulation. Interactions between the geminiviral proteins RepA or Rep and the host cell cycle regulator pRBR have been the most extensively studied. The RepA protein of mastreviruses, which is a replication enhancer, interacts with pRBR via a conserved LxCxE motif (79 – 84). In begomoviruses that do not encode a RepA protein, it is the Rep protein that can directly interact with pRBR (85). The Rep protein of begomoviruses lacks the LxCxE motif, and the interaction with pRBR takes place in the middle of an alpha-helical motif located in the N-terminal region (86, 87). Geminivirus Rep proteins have also been found to interact with other host proteins involved in cell cycle regulation, as, for example, with the cellular proliferating cell nuclear antigen (PCNA) (88, 89), SUMO conjugation enzymes (89 – 91), a protein kinase, and the histone H3 (92). Interactions between other geminivirus proteins and host proteins have been identified, as, for example, the geminiviral replication enhancer REn and PCNA (88), REn and pRBR (93) or C4 and a ubiquitin E3 ligase KRP (94), phosphorylation and SHAGGY-like kinases (95), and the NbSKη kinase (96).

#### Complementary-strand synthesis

The first step of replication is the synthesis of the complementary-strand DNA (csDNA) to convert the viral ssDNA entering the host cell into a dsDNA on which replication will proceed. By studying incomplete intermediates of csDNA synthesis from leaves infected by the african cassava mosaic virus (ACMV; *Begomovirus*, bipartite), Saunders and colleagues (32) determined that the csDNA synthesis is initiated into the CR, near a sequence that can potentially form a stem-loop structure. The csDNA intermediates are associated with RNA moieties, suggesting that csDNA synthesis may be preceded by the synthesis of an RNA primer (32).

Several studies consistently showed that small DNA fragments are associated with mastrevirus particles and are able to prime the csDNA synthesis *in vitro* (25, 26, 28 – 31). These DNA primer molecules are complementary to the small intergenic region (SIR) of the viral-strand DNA. They are mainly about 80 nucleotides but heterogeneous in length with a conserved 5′-end position and a variable 3′-end position. Their 5′-end is linked to a few ribonucleotides which are supposed to derive from a larger RNA.

These results, obtained partly on mastreviruses and partly on begomoviruses, together suggest that the csDNA synthesis is initiated into a conserved non-coding region (SIR for mastreviruses or CR for begomoviruses) *via* the synthesis of an RNA primer probably synthetized by a host primase. Recently, Wei and Lozano-Durán (33) investigated the role of the primase subunits PRIM1 and PRIM2 of the DNA polymerase α which is required for complementary-strand synthesis (see below). They determined that silencing of *PRIM1* or *PRIM2* genes in *Nicotiana benthamiana* virtually abolished the accumulation of the monopartite begomovirus tomato yellow leaf curl virus (TYLCV). However, the authors cautiously indicated that this effect could be due to a destabilization of the DNA polymerase α caused by the absence of one of its subunits, and that the direct involvement of primases, thus, awaited confirmation. In this direction, a quantitative trait locus analysis determined that mutations in the *DNA Primase Large subunit* (*PRiL*) gene of *Cucumis melo* genome could confer resistance to the tomato leaf curl New Delhi virus (ToLCNDV) (34). In the same study, the silencing of *N. benthamiana PriL* led to a severe reduction of accumulation of different geminiviruses, further supporting a role of primase subunits in the initiation of complementary-strand synthesis.

In some cases, and for unknown reasons, the csDNA synthesis could stop soon after its initiation (about 80 nucleotides), resulting in viral ssDNA molecules with a small double-stranded region as those found in mastreviruses virions. These partially dsDNA molecules could be encapsidated and moved to a new cell or host. The small complementary DNA fragments could then prime csDNA synthesis during a new infection event after uncoating of the viral DNA (30). However, it is unclear whether this scheme applies to all geminiviruses since no DNA primers similar to those of mastreviruses were found associated with ACMV virions (97).

As the geminivirus genome does not encode a polymerase, the synthesis of the csDNA is performed by a cellular DNA polymerase complex. It has been demonstrated that polymerase α is essential for geminiviruses csDNA synthesis (35). Nevertheless, due to its low processivity, the authors did not exclude the involvement of another polymerase yet to be identified. Likewise, the possible involvement of translesion synthesis polymerases (TLS; *Polζ* and *Polη*) has been mentioned (98). These enzymes are specialized DNA polymerases that can replicate damaged DNA templates by bypassing DNA lesions and have recently been shown to stabilize the genome of human dsDNA herpesvirus (99). The euphorbia yellow mosaic virus (EuYMV) could infect *Arabidopsis thaliana* lines knocked out for these genes, but deep sequencing revealed a significantly enhanced mutation rate. The authors concluded that TLS are not mandatory but that they may contribute to complementary strand synthesis, particularly at very early stages of infection (98).

Following the synthesis of the csDNA, the viral DNA is in a circular double-stranded form.

#### Recognition of the viral DNA by the Rep protein

The replication of the genome of geminiviruses is initiated by Rep proteins. They are not DNA polymerases, but a family of replication initiator proteins, promoting or controlling the RCR mechanism (100). Rep proteins bind the viral single- or double-stranded DNAs at the CR in a sequence-specific manner (37, 38, 54, 55). The CR, which is variable among geminiviruses but identical between components DNA-A and DNA-B of bipartite begomoviruses, contains two key elements for replication: short iterative sequences called iterons and an inverted complementary sequence that can form a stem-loop structure.

Several studies have demonstrated that the specific recognition between viral genomic DNA and Rep protein is driven by iterons (Table 1). A comparative analysis of the intergenic regions from 30 geminiviruses (36) identified short iterative sequences of various sizes (Fig. S1A). The arrangement of these motifs is highly conserved among the different phylogenetic clades of geminiviruses, but their sequences are specific to viral species. The Rep protein of the tomato golden mosaic virus (TGMV) binds with high affinity to a GGTAGTAAGGTAG sequence, composed of two GGTAG motif repeats separated by TAA, located in the CR upstream of the sequence potentially forming a stem-loop structure (56). The authors performed competition assays between the complete repeated sequence ( GGTAGTAAGGTAG ), the 5′-motif unit plus the spacer (GGTAGTAA), and the spacer plus the 3′-motif unit (TAAGGTAG). They determined that the Rep protein recognizes the combination of the two repeats. Rep binds preferentially to the 3′-motif, but both repeats are necessary for high affinity. The Rep affinity is reduced by the replacement of the first two guanines by cytosines in the repeated motif and much more so when these mutations are engineered in the 3′-repeat (GGTAGTAAccTAG) than the 5′-repeat (ccTAGTAAGGTAG). The same authors further investigated the importance of these iterons in viral replication by transfecting a fragment of the TGMV DNA-A sequence containing wild-type (GGTAGTAAGGTAG) or mutated (ccTAGTAAGGTAG; GGTAGTAAccTAG) motifs with a Rep gene (*AL1*) expression vector in *Nicotiana tabacum* protoplasts. No newly synthetized DNA replicon accumulation was detected for the 3′-mutated iteron (GGTAGTAAccTAG). Low level of viral accumulation was detected for the 5′-mutated iteron (ccTAGTAAGGTAG) and solely when a vector expressing the replication-activating gene *AL3* was added but much lower than the wild-type sequence. Thus, the two iterons are involved in recognition by Rep, but they do not have the same importance for viral replication. Additionally, the importance of the distance between the iterons and the stem-loop sequence was highlighted by a study on TGMV, where the addition of 7, 10, or 13 nucleotides between the two almost completely abolished viral replication, whereas the addition of only three nucleotides at the same location had little effect (57). The authors demonstrated that the deleterious effect on the replication of the additional sequences was caused by the alteration of the iteron/stem-loop distance rather than by the nature of the inserted sequence. Though most studies located the iteron sequences of the members of the genus *Begomovirus* on the left side of the stem-loop sequence, it was recently demonstrated that the mung yellow mosaic India virus (MYMIV; *Begomovirus,* bipartite) genome has iteron sequence CGGTGTA repeated on both sides of the stem-loop sequence with two repeats on the left and one on the right (53). Moreover, studies conducted on two mastreviruses, the wheat dwarf virus [WDV; (46, 47)] and the maize streak virus [MSV; (50)] also showed Rep-binding sites on both sides of the stem-loop sequence.

Fontes and collaborators (39) analyzed the binding specificities of the two bipartite begomoviruses TGMV and bean golden mosaic virus (BGMV) Rep proteins *in vitro*. For this purpose, the authors swapped the iteron sequences of the TGMV and BGMV DNA-B components and analyzed the ability of the Rep protein of each virus to bind to the wild-type and mutated DNA-B. The TGMV Rep protein was able to bind to the mutated BGMV DNA-B component containing the iteron sequence of the TGMV, while the BGMV Rep protein did not. Conversely, the BGMV Rep protein bound to the mutated TGMV DNA-B containing the BGMV iteron sequences, while the TGMV Rep protein did not. Despite efficient Rep fixation observed *in vitro*, no replication of the DNA-B mutants was observed when they were co-infiltrated with wild-type DNA-A of each virus in tobacco protoplasts. The authors concluded that Rep binding to iteron sequences is necessary for replication but not sufficient. As the loop sequences of TGMV and BGMV differ by two nucleotides, the same authors then investigated the role of the stem-loop sequence in the recognition specificity of the viral DNAs by their cognate Rep protein. The authors mutated the BGMV DNA-B stem-loop sequence such that it corresponds to the TGMV DNA-B sequence. They then analyzed the accumulation of wild-type and mutated BGMV DNAs-B when co-transfected with BGMV or TGMV DNAs-A in tobacco protoplasts. They observed that the mutated BGMV DNA-B, which has the TGMV stem-loop sequence, is accumulated at a similar level to that of the wild-type BGMV DNA-B in the presence of BGMV DNA-A. No replication was detected when transfected with TGMV DNA-A. The observed sequence variation in the stem-loop sequences is, thus, not involved in recognition specificity of the viral DNA by the corresponding Rep protein.

Proteins of the rep family have three major conserved functional domains: endonuclease and DNA-binding, helicase, and oligomerization (1). While the helicase and endonuclease functions are evoked later in the step-by-step description of the replication process, the DNA-binding motif is important at the very early phases. Specific recognition of the CR sequence by the protein Rep is controlled by the Rep N-terminal region (40, 44). A comparison of the iterons and the N-terminal region of the Rep protein of over a hundred geminiviruses identified an “iteron-related domain” (IRD) (101). This domain results from the assembly of Rep oligomers (55, 61) where a not yet delineated stretch of N-terminal amino acids is folded into a variable structure (102). Amino acid variations in this structure correlate with variations within the iteron sequences, suggesting that the IRD is an important Rep determinant of the specific recognition and replication initiation of a viral ssDNA circle (101, 103).

#### Replication initiation

**Fig 2:**
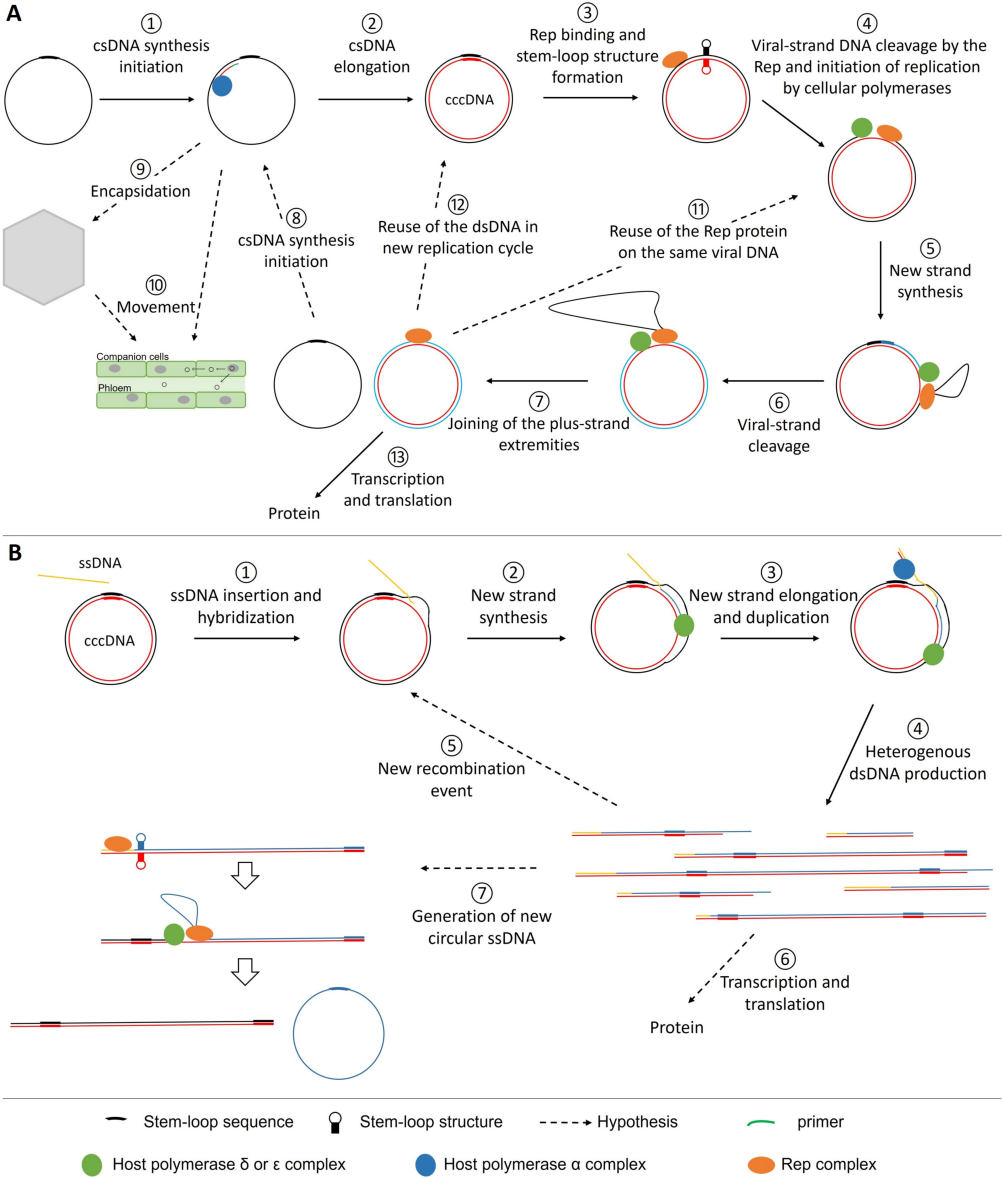
Replication modes of *Geminiviridae* and *Nanoviridae*. (**A**) Schematic representation of RCR of geminiviruses and nanoviruses. First, the complementary strand synthesis is initiated from a primer in a conserved region (SIR in mastreviruses, CR in begomoviruses; CR-M in babuviruses) of a viral single-stranded DNA (1). The elongation of the csDNA is processed by the host cellular machinery, notably polymerase α, and results in a circular covalently closed DNA molecule (cccDNA) (2). The Rep complex, constituted by Rep proteins assembled as oligomers, binds to the iteron sequences. The binding leads to a local melting of the dsDNA favoring the formation of a stem-loop structure (3). This stem-loop structure exposes a nanonucleotide single-stranded loop and permits the preferential cleavage of the positive strand by the Rep complex. Rep remains linked to the 5′-end of the cleaved strand and the 3′-OH extremity serves as primer for the host polymerase (4). Rep and the polymerase move through the viral DNA. With its helicase activity, Rep unwinds the dsDNA and the polymerase synthesizes the new positive strand using the complementary strand synthesized in step 1 as a template (5). The positive strand is cut by the Rep in the conserved nonanucleotide sequence, thus separating the replicated strand (black strand) from the newly formed strand (blue strand) (6). The two extremities of the replicated positive strand are joined by the Rep to form a full-genome length circular DNA (7). On the one hand, a new csDNA initiation event can occur on the released circular ssDNA [black; (8)]. The csDNA can be completely synthetized and enter a new replication cycle (2). The elongation of csDNA could also be stopped prematurely and the generated DNA molecules could be encapsidated (9) and/or move outside of the cell (10). On the other hand, the dsDNA (7) could be used in a new replication cycle, either directly by recruiting a new polymerase complex (11) or by recruiting a new Rep protein (12). It could also be used to produce mRNAs and viral proteins (13). (**B**) Schematic representation of recombination-dependent replication of geminiviruses. First, a viral ssDNA fragment (yellow) is inserted between the two strands of a cccDNA (1). The ssDNA fragment hybridizes with the homologous region inside the cccDNA and primes the synthesis of a new strand (blue) along the cccDNA template (2). During elongation, the new synthetized strand is converted into dsDNA by a host polymerase complex (3). The RDR mechanism leads to the formation of linear dsDNA molecules of various sizes (4). The complementary strand of some of them is not fully synthetized and their 3′ overhang can initiate new RDR events (5). The linear dsDNA can be transcribed to produce viral proteins (6). Some of these linear dsDNAs contain several origins of replication and can serve as replication templates by Rep to produce a circular ssDNA in a way similar to RCR (7).

The stem-loop sequence being highly conserved in the non-coding region (LIR/IR) of all geminiviruses, its role in replication has been further investigated (36, 39, 67). This sequence contains a central loop-forming nonanucleotide motif, TAATATTAC for most geminiviruses, or TAAGATTCC for *Eragrovirus* and *Becurtovirus* (7), flanked by reverse complementary sequences constituting the stem that can vary according to the viral species (39, 67). Located into the highly conserved CR, the stem-loop sequence of bipartite begomoviruses is identical between DNA-A and DNA-B. The Rep protein has a DNA cleavage activity between nucleotides T and A in bold of the nonanucleotide sequence TATTAT**T/A**C of the TYLCV (*Begomovirus*, monopartite) (67). Nucleotide substitutions in this sequence (including changes of the nucleotides T and A in bold) did not affect the location of Rep cleavage, which remained between nucleotides seven and eight of the loop sequence but decreased its efficiency (i.e., the cleaved DNA quantity) (67). Contradictory to the fact that the viral DNA is double-stranded at this early stage of the replication cycle, the TYLCV Rep protein cleaves only single-stranded DNA and preferentially the positive one. The single-stranded negative strand can also be cleaved between the nucleotides in bold TAATAT**T/A**TA or TAATATTA**T/A**, but the products are detected only after a long exposure time, indicating the lower efficiency of the reaction (67). The same authors (67) reported an efficient cleavage of oligonucleotides lacking the flanking sequence on either side of the loop-forming nonanucleotide motif and concluded that the stem structure is not required. However, Orozco and Hanley Bowdoin (62) showed that the stem-loop structure formation is essential for TGMV replication *in vivo*. Most likely, the important role of the stem, which forms in mirror (Fig. 2A, step 3), is to force the separation of the two strands of the loop-forming nonanucleotide and, thus, render the Rep cleavage site accessible as single-stranded. Consistently, Singh and colleagues (53) indicated that MYMIV Rep binding to the iterons, located on both sides of the stem-loop sequence, distorts the dsDNA at the origin of replication (ORI). It is noticeable that a similar observation was reported for pT181 plasmid, which replicates in a rolling circle manner and where the RepC protein binding enhances the formation of the stem-loop structure *in vitro* and probably *in vivo* (67, 104). Together, these results suggest that the fixation of the Rep protein on the geminiviruses dsDNA ORI sequence induces the formation of the stem-loop structure. Rep is then able to efficiently cleave the positive single-stranded nonanucleotide loop, leaving the negative csDNA mostly uncleaved.

The sequence of the stem appears to be important for its facilitating role in loop-forming. Indeed, Orozco and Hanley-Bowdoin (62) introduced paired-mutations in order to change the sequence of the stem without affecting the structure formation in TGMV DNA-B. These modifications impaired DNA-B replication when expressed with a competing wild-type origin provided by DNA-A *in vivo*. An explanation could be that, although they do not prevent the formation of the stem-loop structure, these mutations could affect the stability of base-pairing and, thus, making it less competitive than the wild-type sequence. Consequently, and although the precise series of events initiating replication remains partly unclear, it likely involves both the stem-loop structure and the nature of the sequence composing it.

#### Cleavage, elongation, and joining

After viral DNA recognition and cleavage in the conserved nonanucleotide sequence, Laufs and colleagues (67) showed that the TYLCV Rep protein remains covalently linked to the 5′-extremity of the cleaved positive single-strand. According to the replication model proposed by these authors, the released 3′-extremity is then available to prime the synthesis of the new plus-strand, while the 5′-end linked to the Rep is displaced along the viral DNA. It has been demonstrated recently that the host DNA polymerase δ is recruited by the C3 replication enhancer protein of different geminiviruses to synthetize the new viral DNA strand (35). In C3 null mutants, it is the host polymerase ε which allows the synthesis of the new viral ssDNA.

Once the full-length DNA is polymerized, the newly synthetized nonanucleotide sequence is recognized and cleaved by the Rep protein. Finally, the 3′- and 5′-ends of the new synthetized DNA are joined by the Rep, constituting a free circular single-stranded DNA (67).

Although viral replication is ATP-dependent, the cleavage and ligation steps are not (67). The same study further suggested that the Rep protein may have helicase activity requiring ATP, like some Adeno-associated viruses. Consistent with this speculation, a helicase activity was later directly demonstrated *in vitro* for the Rep of the tomato yellow leaf curl Sardinia virus (TYLCSV, *Begomovirus*, monopartite) (71) and *in planta* for that of MYMIV (72). This latter study also highlighted a similar *in vitro* helicase activity for two additional begomoviruses, respectively, close to (mung bean yellow mosaic virus) and more distant from (Indian cassava mosaic virus) MYMIV, suggesting that the helicase activity could be conserved among all geminivirus Rep proteins. Both studies showed that the geminivirus helicase activity is dependent on Rep proteins oligomerization and the unwinding is made from 3′- to 5′-end direction of the template strand.

#### Integrated model for geminiviruses rolling circle replication

Based on the information provided by this literature, a diagram of geminiviruses replication *via* the RCR mechanism is proposed in Fig. 2. First, the csDNA synthesis is primed by an RNA or DNA primer in a conserved region (1). The csDNA elongation is then processed by the host cellular machinery, involving polymerase α (2). The Rep complex, composed of Rep proteins assembled as oligomers, binds the viral DNA at the iteron sequences that can be located either upstream or on both sides of the stem-loop sequence. The iteron sequences confer the specificity of recognition of the viral DNA by its Rep protein. The nucleotide sequence of the iterons itself, that of the flanking sequences, the spacing between the iterons, and the spacing between iterons and stem-loop are all important. The binding of the Rep complex to the iteron sequences likely imposes physical constraints leading to a distortion of the DNA structure at the ORI, locally melting the double-strand and allowing the stem-loop structure formation (3). The formation of this structure exposes a nonanucleotide single-stranded loop, and the positive strand can then be preferentially cleaved by the Rep complex. After cleavage, Rep remains covalently linked to the 5′-end of the cleaved positive strand and the 3′-OH extremity serves as host polymerase primer (4). The synthesis of the new strand is performed by the host polymerases δ or ε depending on whether the replication enhancer protein C3 is respectively present or not, using the csDNA synthesized in (2) as template (5). With its helicase activity, Rep unwinds the dsDNA from the 3′- to the 5′-end of the csDNA to allow the replication complex to progress. Once the new full-length DNA synthetized, Rep cuts the positive strand in the conserved nonanucleotide sequence, thus separating the replicated strand from the newly formed strand (6). Finally, Rep joins the two ends of the replicated positive strand to form a free circular DNA strand (7).

It is likely that a new csDNA initiation event occurs on the released circular ssDNA (8). The synthesis of the csDNA can be done entirely. In this case, the dsDNA can enter a new replication cycle (2). Otherwise, the elongation of csDNA could be stopped prematurely. The DNA molecules, with a small double-stranded portion, would be encapsidated (9) and/or move outside of the cell (10). The dsDNA generated by the RCR mechanism (7) could directly perpetuate the replication cycle by continuing to use the same Rep and polymerase complexes (true rolling circle replication [RCR] [11]), or initiate a new cycle after recruiting new Rep and polymerase complexes (12). The dsDNA is also the form which is transcribed to produce viral RNAs and proteins (13). To our knowledge, the determinants of the distinct pathways and the proportion of the different RCR products following each pathway have not been characterized.

### Nanovirids

Unlike geminiviruses, the replication of nanovirids has been little studied. Since their genome encodes a Rep protein and is constituted of circular genome segments each harboring non-coding regions similar to those described in geminiviruses, it is assumed that their replication also follows a rolling-circle mechanism. The few studies available explore different features of RCR in nanovirids in comparison with geminiviruses. While showing many similarities between the two virus families, they also point at some potential differences. As with geminiviruses, nanovirid replication takes place in the nucleus of the host cell. All nanovirids are restricted to phloem companion cells (105), where they likely also partially reprogram the cell into a replicative phase of the cell cycle. Consistently, the nanovirid Clink protein (for “cell cycle link”) possesses the LxCxE motif allowing its interaction with members of the pRB family and, hence, stimulates viral replication (106 – 108). Clink also interacts with a S-phase kinase-associated protein 1 (KPN1) homolog (107). The nanoviral Rep proteins lack the LxCxE motif, and there are no reports of its direct involvement in host cell cycle manipulation.

#### Complementary-strand synthesis

As for mastreviruses in the family *Geminiviridae*, DNA primers have been shown to be associated with BBTV (*Babuvirus*) segments extracted from purified virions (27). These primers have heterogeneous length, but the majority is about 80 nucleotides. All are initiated within the CR-M with a variable initiation site. Intriguingly, most of these primers extend beyond the end of the CR-M, into a non-conserved region where they show homology only to DNA-C. No ribonucleotides were found linked to the primers, but the authors of the study do not exclude a possible degradation of the RNA during the experimental procedure or removal by unknown processes before encapsidation. These primers are capable to prime csDNA synthesis of BBTV DNA components *in vitro*. Although the authors pointed out that the DNA obtained by virion purification contains similar amounts of each BBTV component, most of the self-primed products were derived from BBTV DNA-C, consistent with the sequences of the primers identified. Products derived from DNA-M, N, R, and U3 were similarly weakly accumulated, but no products were detected for DNA-S. According to the authors, it is likely that DNA-S is capable of self-priming but that the reaction products are too rare to be detected. More recently, it has been shown that BBTV DNA components accumulate at different frequencies in banana plants, and although DNA-C accumulates more than DNA-S, both are poorly accumulated compared to DNA-N, R, and U3 (109, 110). This leaves the observation of an overrepresentation of segment C primers completely unexplained. DNA-C encodes the protein Clink, which interacts with cell cycle regulators to enhance replication (7, 107), and DNA-S encodes the coat protein (111). Typically, during viral infection, genes encoding proteins involved in replication are first expressed. Those encoding structural proteins, such as coat proteins, are expressed later (112). Thus, there may be a link between complementary-strand synthesis (providing transcription template) and gene expression timing, where DNA-C would be preferentially expressed at early stages.

#### Recognition specificity

In nanovirids, it is generally assumed that the recognition specificity of the virus DNA by its Rep protein is also determined by iterons. Iteron sequences are generally conserved between genomic segments of a given species. However, there must be some exceptions since an iteron located on the left side of the subterranean clover stunt virus (SCSV, *Nanovirus*) stem-loop sequence shows two different groups of sequences (DNA-C/M/U1/U4 and N/R/S/U2) (Fig. S1E). The nanovirids iterons have a different arrangement than those of geminiviruses. The sequences are not necessarily repeated in tandem, and some are inverted repeats. In addition, iteron sequences are always found on both sides of the stem-loop sequence unlike for geminiviruses where iteron sequences are often found only upstream of the stem-loop (Fig. S1B through F) (48, 49, 51, 52, 113). Introduction of mutations in some BBTV DNA-N iteron sequences affected its replication in banana embryogenic cells, and the quantitative effect depended on the iteron which was mutated (51). The ability of each of the faba bean necrotic yellows virus (FBNYV), milk vetch dwarf virus (MDV), and SCSV Rep proteins to initiate replication of DNA-S of heterologous species when agro-infiltrated in leaves of *Nicotiana benthamiana* has been studied (49). These three viruses share conserved sequences in the replication origin of their segments, especially FBNYV and MDV (Fig. S1B, D, and E). Their Rep proteins also share similarities in their amino-acid sequences. By measuring segment accumulation in *N. benthamiana* leaves, the authors demonstrated that the Rep proteins of the three nanoviruses can drive the replication of heterologous nanoviruses DNA-S. However, the SCSV DNA-S accumulation is lower with the Rep of FBNYV and MDV than with its cognate Rep. Similarly, accumulation of MDV DNA-S with the SCSV Rep protein is lower than with the MDV or FBNYV Rep. This is perfectly in line with observed iteron sequences, for which FBNYV and MDV are more similar and SCSV more distant (Fig. S1B, D, and E).

Due to both the poor characterization of iterons and the small number of available sequences, whether an IRD functional domain exists in nanovirus Rep proteins and specifically determines the ssDNA recognition and replication initiation is undocumented.

#### Replication of the positive strand

As in geminiviruses, the main conserved region of nanovirids contains a sequence potentially forming a stem-loop structure (Fig. 1B) (15 – 18). The stem sequences vary not only between viral species but also between genome segments of a species. For example, the faba bean necrotic stunt virus (FBNSV, *Nanovirus*) inverted-repeat sequences are identical for DNA-M/N/R/S/U2/U4 and DNA-C/U1 but differ between these two groups of segments. The loop also contains a nonanucleotide motif, conserved between the species of a given genus: AGTAT**T/A**C in *Nanovirus* and ATTAT**T/A**C in *Babuvirus* (48, 64) (Fig. S1). As for geminiviruses, the nanoviruses Rep proteins possess a cleavage and joining activity. Rep cleaves the virion-sense DNA sequence in a single-stranded form, into the nonanucleotide motif between nucleotides T and A in bold (48, 64). After cleavage and during the elongation of the new strand, the Rep protein of BBTV remains covalently linked to the 5′-end of the positive single strand. Once the entire virus strand is replicated, the Rep complex catalyzes the joining of the two extremities of the replicated strand (64).

No studies have yet investigated helicase activity in nanovirids. Nevertheless, the Rep amino-acid sequence of FBNYV has a nucleoside triphosphate-binding motif (GxxGxxGKT/S), which suggests an ATPase activity essential for viral replication (48). By homology with geminiviruses, this ATPase activity could be required for a helicase function of nanovirids Rep proteins, but this has not yet been experimentally demonstrated.

#### Comparison of nanoviral and geminiviral RCR

In summary, nanovirids RCR starts by csDNA synthesis. As established for the mastreviruses of the family *Geminiviridae*, babuviruses have also been shown to encapsidate DNA primers enabling the priming of the csDNA synthesis. These primers match to a non-coding region, near a sequence that could potentially form a stem-loop structure. However, contrasting with geminiviruses and despite a similarity in size, the babuvirus primers have a variable 5′-end which does not seem to be linked to ribonucleotides. Perhaps biologically significant, but yet uncharacterized, these babuvirus primers extend into non-conserved regions conferring them a segment-specificity, which has not been reported in bipartite begomoviruses.

Once the csDNA is synthetized, replication is initiated in a stem-loop sequence located in the conserved region CR-SL, which, as for geminiviruses, can differ in sequence between virus genera and species. A peculiarity of nanovirids is that the stem sequence can also vary between genome components of a species, while it appears to be the same for components DNA-A and DNA-B of bipartite geminiviruses. The differences between stem-loop sequences of distinct segments of a given nanovirus species have thus far not been shown to have any functional significance, a point further discussed later.

In both families, the specificity of recognition of the viral DNA is driven by the iterons. Beyond their slightly different arrangement, the nanovirid iterons have always been identified on both sides of the stem-loop sequence while those of the geminiviruses are located either upstream or on both sides.

Finally, nanovirid Rep protein could possess an ATPase activity that could indicate a helicase function as shown in geminiviruses. Table 1 summarizes the similarities and differences between the RCR replication process of geminiviruses and nanovirids. Though some differences exist, RCR replication for geminiviruses and nanovirids is very similar. Therefore, the diagram presented in Fig. 2A could broadly apply to nanovirids as well.

## RECOMBINATION-DEPENDENT REPLICATION

By studying the replicative intermediates of the bipartite begomovirus ACMV with two-dimensional agarose gel electrophoresis, Saunders and colleagues observed concatemeric linear and totally or partially double-stranded DNA forms not consistent with the RCR mechanism, for which open circular double-stranded DNAs, covalently closed double-stranded DNAs (cccDNA), and linear or circular single-stranded DNAs are expected (20). Later, combining two-dimensional gel electrophoresis and electron microscopy, the group of Holger Jeske (114) determined that only a minority of abutilon mosaic virus (AbMV; *Begomovirus*, bipartite) DNA intermediates are compatible with RCR. Most of the DNA forms that were observed were linear dsDNAs without super-helicity, heterogeneous in length and in 5′- and 3′-ends on the AbMV genome. The authors concluded that these replication intermediates are consistent with the RDR mechanism, which is also found in some phages such as the dsDNA bacteriophage T4 (115). Nevertheless, as the authors pointed out, even if they suggested an RDR for AbMV, they did not completely prove it. After this seminal discovery, the DNA forms consistent with RDR were found in geminiviruses belonging to different genera, suggesting that this replication mechanism could be common in this virus family (116 – 120).

Assuming that this replication mode is indeed occurring, and based on phage replication knowledge, a model for RDR in geminiviruses is presented in Fig. 2B. Since it requires the involvement of cellular factors involved in DNA replication and in DNA damage repair, RDR must take place in the host cell nucleus. First, a viral ssDNA fragment (yellow), probably originated from partially replicated ssDNA, is inserted anywhere between the two strands of a cccDNA generated by the synthesis of the csDNA (1). The ssDNA fragment hybridizes with the homologous region inside the cccDNA and primes the synthesis of a new strand (blue) along the cccDNA template (2). The elongation of the newly synthetized ssDNA continues along the circular double-stranded teplate during one or more rounds, possibly processed by host polymerase δ, as shown for step 5 of RCR (Fig. 2A) (35). During the new strand extension, the virus-strand (yellow then blue) is converted into dsDNA by a host polymerase complex (114, 121) (3). This step probably involves host polymerase α since Wu and colleagues (35) determined that this enzyme is required to generate double-stranded intermediates in geminiviruses (Fig. 2A, step 1). RDR leads to the formation of linear dsDNA molecules of distinct sizes and different 5′- and 3′-ends according to the site of initiation and ending of elongation (4). The factor determining elongation termination is not known. Sometimes, the csDNA is not fully synthetized, and some extremities remain single-stranded. The 3′-overhang generated by these incomplete syntheses could be used to initiate new RDR events (5) (121). The linear dsDNA could be transcribed to produce viral proteins (6). Some of them, generated after several rounds around the circular DNA template, possess multiple origins of replication and could be used as replication templates by Rep to produce a circular ssDNA (121) (7). Thus, the RDR mechanism does not use the ORI and does not involve the Rep protein but for step 7. Interestingly, without this last step, the whole RDR process would not be a mode of replication but perhaps a way to produce transcription templates. More generally, in the absence of a better understanding of the processes controlling RDR, it is difficult to appreciate the fate of its products and, hence, its role in the viral cycle functioning.

As earlier reported (122), geminivirus infection generates a genotoxic response activating the host DNA-repair machinery, promoting somatic homologous recombination (78, 123), which most likely induces the RDR mechanism. The TLS Polζ and Polη, mentioned in a previous section for a possible role during complementary strand synthesis, may also contribute to RDR (98). Most interestingly, a series of studies have highlighted the role of recombination mediator proteins in the replication of distinct geminiviruses. A phage display screen for *Arabidopsis thaliana* proteins binding to the mung bean yellow mosaic India virus (MYMIV) Rep identified RAD54 (124) and RAD51 (125). In these studies, the interactions were confirmed using yeast two-hybrid and co-immunoprecipitation assays, and the requirement for replication was supported by *ex vivo* approaches either in yeast or in infiltrated plant leaves monitoring geminivirus DNA replication in the presence/absence of RAD54 or RAD51. Two complementary studies further investigated the role of RAD54 (126) and several paralogs of RAD51 (127) in geminiviral infection. These *in vivo* studies used *Arabidopsis* gene knockouts challenged with EuYMV. While the involvement of RAD54 could not be confirmed *in vivo*, the paralog RAD51D proved to promote viral replication at the early stages of infection. This activity was further shown to be linked to RAD51D earlier reported single-strand annealing recombination capacity (128), indicating a putative important role in both the formation of replicative intermediates and the RDR process (127).

Viral intermediates from both RCR and presumed RDR are produced from the early stages and simultaneously during systemic infection of leaves with a geminivirus (114, 117, 129). These observations are not consistent with the replication mechanism of T4 phage for which the two mechanisms occur sequentially: RCR occurs in the early stages of infection; then, once viral accumulation reaches some threshold, the replication mode switches to RDR (117). The authors proposed that the apparent simultaneous production of intermediates of both replication modes could result from several asynchronous geminivirus cell infection events in the leaf tissues.

Thus far, no analogous study has been conducted for nanovirids, and whether they could also use RDR for viral DNA replication is totally unknown.

## CONCLUDING REMARKS

Numerous studies on geminivirus replication have allowed a good characterization of the RCR mechanism in these ssDNA viruses and permitted to establish a detailed diagram of RCR. Two genomic elements are crucial for RCR in *Geminiviridae*: (i) the iteron sequences that determine the specific recognition of the viral DNA by its Rep protein and (ii) the stem-loop sequence and structure that constitute the ORI. The few studies conducted in *Nanoviridae* suggest an RCR mechanism broadly similar to that of *Geminiviridae*. Furthermore, some intriguing observations indicate the possible existence of a second mode of replication by RDR. In contrast to RCR, this potential replication mode has been poorly studied in *Geminiviridae,* and no studies have been conducted in *Nanoviridae*, leaving this process poorly understood.

Related to the multipartite architecture of nanovirids, many aspects of their life cycle remain enigmatic (130, 131), and replication is one of them. In addition to having their genome divided into a large number of genome segments, each of them accumulates at different relative frequencies within host plants. The frequency pattern of the distinct segments accumulating within infected plants, designated the genome formula, is specific to the host species (132). It was recently shown (133) that the host-related changes of FBNSV genome formulas are not induced by sequence modification. The same study highlighted a link between FBNSV genome formula and gene expression, suggesting that genome formula variations could allow FBNSV to adjust the expression of its genes upon host-switch by modulating their copy number. To date, the mechanisms of establishment and regulation of the genome formula are unknown. An obvious question is whether a differential replication of the distinct genome segments could explain their differential accumulation within the host plant. First, it is unlikely that differential segment accumulation results from different affinities with the replication complex because the iteron sequences allowing the recognition of the viral DNA by the Rep complex are highly conserved between different segments of the same nanovirus species. Second, the sequences of the stem allowing the formation of the stem-loop structure, hence permitting viral strand cleavage by the Rep complex, differ among segments. These differences could potentially affect replication efficiency by varying the distance between the Rep-binding site (iterons) and the cleavage site (loop) or by affecting the stem-loop structure stability. However, this cannot provide a satisfactory explanation either because in the two groups of FBNSV segments M/N/R/S/U4 and C/U1, each containing segments with the exact same stem-loop sequence [Fig. S1C; (134)], some are highly accumulated (e.g., N or U4 in *Vicia faba*) and others are rare [e.g., R and S in *V. faba;* (132, 133)]. Third, RDR could occur preferentially on certain segments, which would generate differential accumulations. Again, this does not appear as a satisfactory explanation, as it could hardly explain the genome formula changes upon host-switching. Finally, the initiation of the csDNA synthesis is a crucial step for replication since it allows the production of template for RCR and RDR. Interestingly, from the study by Hafner and colleagues (27), it appears that the synthesis of the BBTV csDNA is not equally primed over the six components *in vitro,* and this could be another explanation for the unequal accumulation of the segments. In this study, most self-priming extension products corresponded to DNA-C, whereas no DNA-S extension could be detected. Different efficiency of primer association and encaspidation with distinct ssDNA segments could result in different production of csDNA early in infection and, consequently, in later accumulation building up the genome formula. This possibility has not been tested experimentally, and a parallel quantification of the efficiency of self-priming and of the genome formula in the same nanovirids species may provide an answer.

While the importance of RDR relative to RCR in the production of viral progeny remains unclear, RDR provides an interesting potential mechanism for the regulation of gene expression that has not been envisaged thus far. The large amounts of linear dsDNA produced by RDR are heterogeneous in length. They can be smaller or larger than the full-length genome, the larger ones appearing as a size continuum rather than as various size classes each corresponding to a round number of full-length genome units. The accumulation of these heterogeneous dsDNAs should, thus, lead to an imbalance in the copy number of the different viral genes, as they are not equally present on these molecules. That the copy number of a gene directly impacts its expression level is extensively documented in all organisms, including nanoviruses [(133) and references within]. Therefore, both the probable distinct relative frequency of the different genes in this RDR-generated population of geminiviral dsDNA, and the fact that they can be transcribed, most likely impacts on gene expression patterns. One could then speculate on a functional role of RDR, other than replication. By analogy to the functional role of the genome formula of the nanovirus FBNSV (133), RDR could be a way to implement amplification-mediated gene expression tuning (135) in geminiviruses, in both monopartite and bipartite species.

This hypothesis, however, should be considered with further nuances. First, transcriptional regulation of gene expression by viral and host proteins has been reported in several instances, imposing that the gene copy number variations could only be one additional regulation level. For example, TGMV Rep protein transcriptionally represses its own gene (136), the transcription of the downstream TrAP and REn genes depends on the suppression of Rep transcription (137), and the CP gene expression may be regulated in several geminivirus species through a conserved transcription factor-binding site (137 – 140). It is here relevant to note that, in such a situation of a transcriptional regulatory network connecting several genes, even minute copy number variations are predicted to have dramatic non-linear positive or negative effects on gene expression, potentially inducing bifurcation in the behavior of the transcriptional regulatory network (141). Second, because the linear dsDNA products of RDR are methylated (142), the genes they contain might not be Pol-II-transcribed into mRNA. However, possible transcription of these RDR products by RNA pol-II has been envisaged (121). Pol-II transcription depends on the number and location of methylated sites, and this issue has not been addressed yet specifically for viral linear dsDNA (142, 143), leaving the question open for future investigation.

## SUPPLEMENTAL MATERIAL

Fig. S1 mbio.01692-23-s0001.docx Alignment of iterative sequences of geminiviruses and nanovirids.

ASM does not own the copyrights to Supplemental Material that may be linked to, or accessed through, an article. The authors have granted ASM a non-exclusive, world-wide license to publish the Supplemental Material files. Please contact the corresponding author directly for reuse.
