# Supplementary material for: Replication mechanisms of circular ssDNA plant viruses and their potential implication in viral gene expression regulation
Source: mBio. 2023 Sep 11;14(5):e01692-23. doi: 10.1128/mbio.01692-23 (PMC10653810; doi:10.1128/mbio.01692-23)
Supplement: Fig. S1 — Alignment of iterative sequences of geminiviruses and nanovirids. [file mbio.01692-23-s0001.docx]

**A**

Loop

| TYLCV-S  TGMV-A  TGMV-B | TGAATCGGGGGACACTCAAAGTATCCAGCAATTGGGGGAATTGGGGGGCAATATATATGATGCCCCCTAAAT---CCGTATAATATT AC  65 nt  Loop  TACTACCAATTG---GAATTGGTAGTAAGGTAGCTCTTATATATTAGAAGTTCCTAAGGGGCACGTGGCGGCCATCCGTTTAATATT AC  TACTACCAATTG---GTATTGGTAGTAAGGTAGCTCTTATATATTAGAAGTTCCTAAGGGGCACGTGGCGGCCATCCGTTTAATATT AC  23 nt  Loop |
| --- | --- |
| MSV | CCGGACCGGTCCGGCCCAGCAGGAAAAGAAGGCGCGCACTAATATT ACCGCGCCTTCTTTTCCTGCGA |

**B** FBNYV

a

a

a

a’

Loop

Stem

Stem

a’

a’

| DNA-C  DNA-M  DNA-N  DNA-R  DNA-S  DNA-U1  DNA-U2  DNA-U4 | GCTGACGTCAGCTGACTCCTTGATGACGTAG..GGGGCGGGGCATAGTATT ACCCCCGCCCC..GGGTTCAG...AGTCACGTACGGAGTG  TGTGACGTCACTTGATCCCTTGCTGAG....CTGGGGCGGGGCTTAGTATT ACCCCCGCCCCAG..GTTCAGCGGAGTCATCACGTGAGAC  TGTGACGTCACTTGATCCCTTGCTGAG....CTGGGGCGGGGCTTAGTATT ACCCCCGCCCCAG..GTTCAGCGGAGTCATTTAGACGTAA  CGTGACGTCATGTGATCCCTTGCTGAG....CTGGGGCGGGGCTTAGTATT ACCCCCGCCCCAG..GTTCAGCGGAGTCATCACGTGGATT  TGTGACGTCACTTGATCCCTTGCTGAG....CTGGGGCGGGGCTTAGTATT ACCCCCGCCCCAG..GTTCAGCGGAGTCATTTAGACGTAA  GCTGACGTCAGCTGACTCCTTGATGACGTAG..GGGGCGGGGCATAGTATT ACCCCCGCCCC..GGGTTCAG...AGTCACGTACGGAGTG  TATGACGTCACTTGATCCCTTGCTGAG....CTGGGGCGGGGCTTAGTATT ACCCCCGCCCCAG..GTTCAGCGGAGTCATCACGTGAGAC  TGTGACGTCACTTGATCCCTTGCTGAG....CTGGGGCGGGGCTTAGTATT ACCCCCGCCCCAG..GTTCAGCGGAGTCATTTAGACGTAA |
| --- | --- |

a’

Stem

Loop

Stem

a’

a

a

a

a’

**C** FBNSV

| DNA-C  DNA-M  DNA-N  DNA-R  DNA-S  DNA-U1  DNA-U2  DNA-U4 | CAGCTGACGTCAGCTGATCCCGTGATGACGTA..GGGACGGGGCTTAGTATT ACCCCCGTCCC..GGGTTCAG...AGTCACGTACGGAGTG  AGCATGACGTCATTTGATCCCGTGCTGAG...CTGGGGCGGGGCTTAGTATT ACCCCCGCCCCAG..GTTCAGCGGAGTCATCACGTGAGAC  AAGATGACGTCATTTGATCCCGTGCTGAG...CTGGGGCGGGGCTTAGTATT ACCCCCGCCCCAG..GTTCAGCGGAGTCATTGAGACTCCA  CGCATGACGTCATTTGATCCCGTGCTGAG...CTGGGGCGGGGCTTAGTATT ACCCCCGCCCCAG..GTTCAGCGGAGTCATCACGTGAGAC  TATGTGACGTCATTTGATCCCGTGCTGAG...CTGGGGCGGGGCTTAGTATT ACCCCCGCCCCAG..GTTCAGCGGAGTCATTGAGACTCCA  CAGCTGACGTCAGCTGATCCCGTGATGACGTA..GGGACGGGGCTTAGTATT ACCCCCGTCCC..GGGTTCAG...AGTCACGTACGGAGTG  AGCATGACGTCATTTGATCCCGTGCTGAG...CTGGGGCGGGGCTTAGTATT ACCCCCGCCCCAG..GTTCAGCGGAGTCATCACGTGAGAC  CTTGTGACGTCATTTGATCCCGTGCTGAG...CTGGGGCGGGGCTTAGTATT ACCCCCGCCCCAG..GTTCAGCGGAGTCATTGAGACTCCA |
| --- | --- |

a

a

a

a’

a’

Loop

Stem

Stem

a’

**D** MDV

| DNA-C  DNA-M  DNA-N  DNA-R  DNA-S  DNA-U1  DNA-U2  DNA-U4 | GTCAGCTGACGTCAGCTGACTCCTTGATGACGTA..GGGGCGGGG.CTTAGTATT ACCCCCGCCCC..GGGTTCAG...AGTCACGTACGGA  ACTTAGTGACGTCATATGATCCCTTGCTAAG...CTGGGGCGGGG.CTTAGTATT ACCCCCGCCCCAG..GATCAGCGGAGTCATCACGTGA  TAATTGTGACGTCATTTGATCCCGTGCTGAG...CTGGGGCGGGG.CTTAGTATT ACCCCCGCCCCAG..GATCAGCGGAGTCATTTAGACT  TATGCATGACGTCATATGATCCCTTGCTGAG...CTGGGGCGGGG.CTTAGTATT ACCCCCG.CCCAG..GATCAGCGGAGTCATCACGTGA  ACCTTGTGACGTCATTTGATCCCGTGCTGAG...CTGGGGCGGGGGCTTAGTATT ACCCCCGCCCCAG..GATCAGCGGAGTCATTTAGACT  GTCAGCTGACGTCAGCTGACTCCTTGATGACGTA..GGGGCGGGG.CTTAGTATT ACCCCCGCCCC..GGGTTCAG...AGTCACGTACGGA  GTCAGCTGACGTCAGCTGACTCCTTGATGACGTA..GGGGCGGGG.CTTAGTATT ACCCCCGCCCC..AGGATCAGCGGAGTCATTTAGACT  ACCTTGTGACGTCATATGATCCCGTGCTGAG...CTGGGGCGGGG.CTTAGTATT ACCCCCGCCCCAG..GATCAGCGGAGTCATCACGTGA |
| --- | --- |

a’

b’

a

a’

b’

a

b

Loop

Stem

Stem

**E** SCSV

| DNA-C  DNA-M  DNA-N  DNA-R  DNA-S  DNA-U1  DNA-U2  DNA-U4 | TATGAC.GTCAT...ATGTCTCCGTGCCTACGTCA.GGGCGGGGCTTAGTATT ACCCCCGCCCC.GGGATCAGAGACATT...TGACCAA  TATGAC.GTCAT...ATGTCTCCGTGCCTACGTCA.GGGCGGGGCTTAGTATT ACCCCCGCCC.CGGGATCAGAGACATT...TGACTAA  CGTGAGAGTCACGTGATGTCTCCGCGACAGGCT..GGCACGGGGCTTAGTATT ACCCCCGTGCC.GGGATCAGAGACATT...TGACTAA  GATGAC.GTCAT...ATGTCTCCGCGACAGGCT..GGCACGGGGCTTAGTATT ACCCCCGTGCC.GGGGTCAGAGACATCACGTGACTCT  TATGAC.GTCAT...ATGTCTCCGCGACAGGCT..GGCACGGGGCTTAGTATT ACCCCCGTGCC.GGGATCAGAGACATT...TGACTAA  TATGAC.GTCAT...ATGTCTCCGTGCCTACGTCA.GGGCGGGGCTTAGTATT ACCCCCGCCCC.GGGATCAGAGACATT...TGACCAA  CGTGAGAGTCACGTGATGTCTCCGCGACAGGCT..GGCACGGGGCTTAGTATT ACCCCCGTGCC.GGGATCAGAGACATT...TGACCAA  GATGAC.GTCAT...ATGTCTCCGTGCCTACGTCA.GGGCGGGGCTTAGTATT ACCCCCGCCCC.GGGATCAGAGACATT...TGACCAA |
| --- | --- |

Loop

Stem

Stem

R

F1

F2

**F** BBTV

| DNA-C  DNA-M  DNA-N  DNA-R  DNA-S  DNA-U3 | AGGGTCCATGTCCCGAGTTAGTGCGCCACGTAAGCGCTGGGGCTTATTATT ACCCCCAGCGCTCGGGACGGGACATCACGTGCAAC  GGGCCAGATGTCCCGAGTTAGTGCGCCACGTAAGCGCTGGGGCTTATTATT ACCCCCAGCGCTCGGGACGGGACATCACGTGCGTC  GGGGAGAATGTCCCGA.........TGACGTAAGCACGGGGGACTATTATT ACCCCCCGTGCTCGGGACGGGACATGACGTCAGCA  AGATCAGATGTCCCGAGTTAGTGCGCCACGTAAGCGCTGGGGCTTATTATT ACCCCCAGCGCTCGGGACGGGACATTTGCATCTAT  GGGCCAGATGTCCCGAGTTAGTGCGCCACGTAAGCGCTGGGGACTATTATT ACCCCCAGCGCTCGGGACGGGACATGGGCTAATGG  CGATGGAGGTTGAATGAACTTCTGCTGACGTAGGCGCTGGGGCTTATTATT ACCCCCAGCGCC.GGGACGGGACATGGGCTTTTTA |
| --- | --- |

**Fig. S1:** Alignment of iterative sequences of geminiviruses (A) and nanovirids (B-F). For geminiviruses, TYLCV-S (genus *Begomovirus*, monopartite) BGMV (genus *Begomovirus*, bipartite) and MSV (genus *Mastrevirus*) sequence alignments are adapted from (36). For the nanovirid species FBNYV (B), FBNSV (C), MDV (D), SCSV (E) and BBTV (F), genome component designations are shown on the left. Sequence alignments are adapted from (41, 49, 51, 113). Inverted repeat sequences (orange horizontal arrows) forming a stem-loop structure are boxed. Vertical arrows indicate the position of cleavage by the Rep protein within the loop. Conserved sequences shared by genome components are also boxed, revealing several putative iterons (named a, a’, b, b’, R, F1 and F2 for nanovirids). For SCSV, there are two different groups of sequences for iteron b’ on the left side of the stem-loop. Black horizontal arrows indicate iteron sequences.
